# Altilix^®^ Supplement Containing Chlorogenic Acid and Luteolin Improved Hepatic and Cardiometabolic Parameters in Subjects with Metabolic Syndrome: A 6 Month Randomized, Double-Blind, Placebo-Controlled Study

**DOI:** 10.3390/nu11112580

**Published:** 2019-10-25

**Authors:** Giuseppa Castellino, Dragana Nikolic, Antonio Magán-Fernández, Giuseppe Antonio Malfa, Roberta Chianetta, Angelo M. Patti, Antonella Amato, Giuseppe Montalto, Peter P. Toth, Maciej Banach, Arrigo F. G. Cicero, Manfredi Rizzo

**Affiliations:** 1 Department of Health Promotion Sciences Maternal and Infantile Care, Internal Medicine and Medical Specialities (PROMISE), University of Palermo, 90100 Palermo, Italy; castellinogiusy@gmail.com (G.C.); draggana.nikolic@gmail.com (D.N.); amaganf@ugr.es (A.M.-F.); chianetta.roberta8@gmail.com (R.C.); angelomaria.patti@unipa.it (A.M.P.); giuseppe.montalto@unipa.it (G.M.); manfredi.rizzo@unipa.it (M.R.); 2 Periodontology Department, School of Dentistry, University of Granada, 18071 Granada, Spain; 3 Department of Drug Science, Biochemistry Section, University of Catania, 95125 Catania, Italy; 4 Department of Biological, Chemical and Pharmaceutical Sciences and Technologies (STEBICEF), University of Palermo, 90100 Palermo, Italy; antonella.amato@unipa.it; 5 Department of Medicine, Ciccarone Center for the Prevention of Cardiovascular Disease, Johns Hopkins University School of Medicine, Baltimore, MD 21205-2196, USA; peter.toth@srfc.com; 6 Department of Preventive Cardiology, CGH Medical Center, Sterling, IL 61081, USA; 7 Department of Hypertension, Medical University of Lodz, 90137 Lodz, Poland; maciejbanach77@gmail.com; 8 Department of Medical and Surgical Sciences, University of Bologna, 40138 Bologna, Italy; arrigo.cicero@unibo.it

**Keywords:** cardiovascular diseases, metabolic syndrome, non-alcoholic fatty liver disease, diabetes mellitus, type 2, cynara, dietary supplements

## Abstract

The objective was to evaluate the effects of 6 months of supplementation with Altilix^®^, containing chlorogenic acid and its derivatives, and luteolin and its derivatives, on cardiovascular risk and hepatic markers in subjects with metabolic syndrome (MetS). A randomized, double-blind, placebo-controlled study was performed in 100 subjects with MetS with a follow-up period of 6 months; 50 subjects were randomized to Altilix^®^ (26 men and 24 women, mean age 63 ± 8 years) and the other 50 to placebo (28 men and 22 women, mean age 63 ± 11 years). Anthropometric, cardiometabolic, and hepatic parameters were assessed at baseline and at the end of follow-up. Carotid intima-media thickness and endothelial function were assessed by doppler ultrasound and by flow-mediated dilation of the brachial artery, respectively. The presence and degree of non-alcoholic fatty liver disease (NAFLD) was assessed by the fatty liver index (FLI), and subjects were divided into three subgroups: (1) without NAFLD; (2) with borderline NAFLD; and (3) with NAFLD. After 6 months of Altilix^®^ supplementation, we found a significant improvement vs. placebo in most of the evaluated parameters, including body weight (−2.40% (95% CI −3.79, −1.01); *p* < 0.001), waist circumference (−2.76% (95% CI −4.55, −0.96); *p* = 0.003), HbA1c (−0.95% (95% CI −1.22, −0.67); *p* < 0.001), plasma lipids, FLI (−21.83% (95% CI −27.39, −16.27); *p* < 0.001), hepatic transaminases, flow-mediated dilation (10.56% (95% CI 5.00, 16.12); *p* < 0.001), and carotid intima-media thickness (−39.48% (95% CI −47.98, −30.97); *p* < 0.001). Further, the improvement in cardiometabolic variables was independent of the degree of hepatic steatosis. Altilix^®^ supplementation improved hepatic and cardio-metabolic parameters in MetS subjects. Altilix^®^ supplementation was a beneficial approach in the management of hepatic and cardiometabolic alterations in MetS subjects.

## 1. Introduction

Metabolic syndrome (MetS) is defined as the coexistence of several risk factors of metabolic origin (with insulin resistance as the usual common pattern) that elevate the risk for cardiometabolic diseases [1]. MetS is a major and escalating public health and clinical challenge worldwide in the wake of urbanization, surplus energy intake, increasing obesity, and sedentary life habits. Its prevalence has increased over time and is now reaching epidemic proportions, with an estimated prevalence of one-fifth of adult populations in western countries [2]. All the cardiometabolic alterations that compound MetS are also key risk factors for the development and progression of non-alcoholic fatty liver disease (NAFLD), that is considered as a hepatic manifestation of MetS [3].

The prevalence of NAFLD reaches alarming proportions, up to 80–90% of obese adults, 30–50% in those with diabetes, and up to 90% in hyperlipidemic subjects [4]. It should be highlighted that liver fat has been associated with poor metabolic health, independent of the obesity state [5,6]. Despite its growing clinical burden, there are no specific drugs for the treatment of these subjects and their management is necessarily related to lifestyle changes. In recent years, there is a growing research focus on prevention and treatment of risk factors associated with both MetS and NAFLD. This includes the use of products of natural origin, also known as nutraceuticals, which can be used as supplements or even replace drugs, particularly in cases of intolerance or when side effects are present [7]. Some nutraceuticals have shown to be effective in the management of different metabolic situations, including MetS and its associated complications, such as NAFLD [8]. They could improve lipid infiltration of the liver but also several other risk factors, including anthropometric, hemodynamic, and biochemical parameters. Considering that NAFLD and cardiovascular diseases have common risk factors, guidelines for the prevention of cardiometabolic diseases make similar suggestions for their prevention and/or management [9], and the role of nutraceuticals cannot be underestimated [10].

*Cynara* spp. are plants that belong to the Asteraceae family that are widely consumed in the Mediterranean region and represent one of the world’s oldest medicinal plants with multiple health benefits due to the high concentration of biophenols [11]. Extracts from different *Cynara* species have shown several beneficial effects, such as anti-inflammatory, hepatoprotective, and lipid-lowering actions, including the ability to inhibit specific liver enzymes [11]. Also, they have proven to be one of the safest nutraceuticals, showing no adverse effects on treated subjects [12]. The extract of *Cynara cardunculus* (L.) subsp. *scolymus* Hayek, known as artichoke, showed an improvement of serum oxidized low-density lipoprotein-cholesterol (LDL) in subjects with MetS [12]. Extract from artichoke leaves has also showed hepatoprotective properties and has been used in subjects with chronic liver disease [13]. In addition, artichoke extract has been proven to inhibit oxidative stress in a dose-dependent way, and cynarine, chlorogenic acid, and luteolin seem to be the most active substances involved in this beneficial antioxidant activity [14]. Previous studies have also shed light on the potential hepatoprotective activity and hypolipidemic effect of *Cynara cardunculus* (L.) in the management of hepatic alterations [15]. It has been shown that luteolin is effective in ameliorating ethanol-induced hepatic steatosis and injury in preclinical studies [16]. This combination of effects induced by this flavone suggests that luteolin may be useful in the control of both NAFLD and cardiovascular risk [17]. In addition, increasing evidence indicates that specific supplements or nutraceuticals have important hepatoprotective roles, improving liver enzymes as well as liver steatosis and its prognosis [18].

Therefore, a hypothesis of the present study was that the supplementation of a *Cynara cardunculus* (L.) subsp. *scolymus* Hayek-based nutraceutical, named Altilix^®^, containing chlorogenic acid and its derivatives, and luteolin and its derivatives, can improve different cardio-metabolic markers in patients with metabolic syndrome (MetS) in a 6 month follow-up study. The primary objective was to assess whether Altilix^®^ can improve different metabolic parameters including body weight, waist circumference, plasma lipids, plasma glycemia, plasma insulinemia, and HOMA (homeostatic model assessment) index, while secondary objectives were to assess whether Altilix^®^ can reduce cardio-metabolic parameters including plasma cytokines (inflammatory markers and adipokines), carotid intima-media thickness, endothelial function, and fatty liver index.

## 2. Materials and Methods 

### 2.1. Design of the Study

A randomized, doubled-blind, placebo-controlled study was designed to test the effects in MetS subjects of Altilix^®^ (Bionap, Belpasso, Catania, Italy) vs. placebo. All subjects were enrolled from the Unit of Diabetes and Cardiovascular Prevention, University Hospital of Palermo, Italy. The study was designed following CONSORT guidelines [19]. Written informed consent was obtained from all participants at enrolment. The procedures adopted in this study were in agreement with the Helsinki Declaration of 1975, as revised in 2013, and were approved by the Research Ethics Committee of the University Hospital of Palermo. The study was also registered with clinicaltrials.gov (NCT03444558).

Inclusion criteria were age >18 years, BMI >25 kg/m^2^, and the presence of MetS according to the International Diabetes Federation (IDF) definition [20]. Diagnosis of MetS was based on the presence of three or more of these criteria: (1) elevated waist circumference (≥94 cm for men and ≥80 cm for women); (2) elevated triglycerides ≥150 mg/dL (1.7 mmol/L) (or drug treatment); (3) reduced high-density lipoprotein-cholesterol (HDL-C) <40 mg/dL (1.0 mmol/L) for men and <50 mg/dL (1.3 mmol/L) for women (or drug treatment); (4) elevated blood pressure systolic ≥130 and/or diastolic ≥85 mm Hg (or antihypertensive drug treatment); and (5) elevated fasting glucose ≥100 mg/dL (or drug treatment). During the study, any dosage from concomitant therapies remained unchanged in order to avoid potential confounders. All subjects were naïve to statin therapy. Exclusion criteria included excessive alcohol use, pregnancy, severe hepatic or kidney disease, serious infections, and malignancies. 

Participants were randomized to receive the Altilix^®^ supplement or placebo through a computer-based random allocation system. The random allocation was performed by an investigator (D.N.) that did not perform the clinical visits. A random ID number was assigned to each participant. Both nutraceutical and placebo pills were provided by Bionap S.R.L. (Belpasso, Catania, Italy) and were identical in size, color, and consistency to ensure blinding of the participants. The supplement (active ingredients: 10–12% chlorogenic acid and derivates and 2–4% luteolin-7-glucoside and derivates) was obtained from leaves of *Cynara cardunculus* (L.) subsp. *scolymus* Hayek. The nutraceutical was prescribed at a stable dose of 150 mg/day for 6 months. Every month, subjects were contacted by phone calls in order to control and reinforce adherence to the study.

### 2.2. Clinical Variables

At baseline, all subjects underwent a medical and physical examination in order to collect clinical data. Body weight and height were measured while all participants were wearing light clothing without shoes with the same body scale. BMI was calculated using the standard formula (kg/m^2^). Waist circumference was measured at the midpoint between the lowest rib and the iliac crest. Blood pressure was measured in the right arm of the seated participants after they emptied their bladder and sat quietly for 5 min, using an automated sphygmomanometer and with an adapted cuff. Blood samples were taken in order to obtain biochemical data. Also, each participant underwent an echo-color doppler ultrasound examination of the carotid arteries, while endothelial function was assessed by flow-mediated dilation of the brachial artery. After 6 months, all variables were re-evaluated. 

### 2.3. Biochemical Analyses

Plasma and serum samples were gathered from each participant at baseline and at the end of follow-up, and some aliquots were stored at −80 °C until analysis if not done immediately. All biochemical variables were measured by routine laboratory methods, including total cholesterol (TC), triglycerides (TG), and high-density lipoprotein-cholesterol (HDL), while low-density lipoprotein-cholesterol (LDL) was calculated using the Friedewald formula. Renal function was assessed by serum creatinine levels and estimation of the glomerular filtration rate was performed. Glycemic-related variables (fasting glycemia and glycosylated hemoglobin (HbA1c)) were also measured, while insulin resistance (IR) and pancreatic β-cell function were assessed using the equations of Matthews et al. [21], according to the homeostatic model assessment (HOMA) (HOMA-IR = (fasting insulin (UI/mL) × fasting glucose (mg/dL))/405) (HOMA-B% = (360 × fasting insulin (UI/mL))/(fasting glucose (mg/dL) – 63)). Hepatic enzymes alanine aminotransferase (ALT), aspartate aminotransferase (AST), and γ-glutamyltransferase (GGT) were also measured. The AST/ALT ratio was calculated as the ratio between the concentrations of the enzymes AST and ALT in the blood. This is a useful index in clinical diagnosis and can also occasionally be elevated in subjects with NAFLD. Fatty liver index (FLI) score was used as marker of NAFLD, with the following formula [22]: FLI = (e 0.953*loge (TG) + 0.139*BMI + 0.718*loge (GGT) + 0.053*waist circumference − 15.745)/(1 + e0.953*loge (TG) + 0.139*BMI + 0.718*loge (GGT) + 0.053*waist circumference − 15.745) × 100. 

The presence and degree of NAFLD was assessed by FLI, and subjects were divided into three subgroups: (1) without NAFLD (e.g., those with FLI < 30); (2) with borderline NAFLD (e.g., those with FLI between 30 and 60); and (3) with NAFLD (e.g., those with FLI > 60). 

### 2.4. Serum Cytokine and Adipokine Quantification

Serum samples were collected from patients and immediately stored at −80 °C until further processing. Secreted cytokine concentrations (ghrelin, GLP-1, IL-1beta, Il-6, leptin, RANTES, resistin) were analyzed by Procarta® Multiplex Immunoassay (Thermo Fisher Scientific Inc., Waltham, MA, USA) using the Luminex MAGPIX instrument (Thermo Fisher Scientific Inc., Waltham, MA, USA). The beads were resuspended in 120 μL phosphate buffer solution and analyzed on a Multiplex MAGPIX system (Thermo Fisher Scientific Inc., Waltham, MA, USA) using the xPONENT 4.1 software for data acquisition. Antibody responses were expressed in median fluorescence intensity (MFI) per sample as stated by manufacturer’s instructions [23].

### 2.5. Color Doppler Ultrasound of Carotid Arteries

B-mode real-time ultrasound was performed at baseline and after 6 months to evaluate carotid intima-media thickness (cIMT). All examinations were performed by a single researcher (A.M.P.) in a blinded manner using the same echograph (SonoAce Pico Ultrasound System, Samsung Medison Co., Korea). The examiner did not have access to previous scans when follow-up studies were performed. The ultrasound examination was performed in a standardized manner with fixed angles of insonation, as previously reported by our group in detail in other studies [24].

### 2.6. Ultrasound Assessment of Endothelial Function

All subjects were banned from drinking coffee or tea and were to avoid smoking 30 minutes before the test. Each subject remained in a supine position for 10 minutes. The same physician (A.M.P.) performed all the ultrasonography tests at the initial visit and after 6 months, in a blinded manner without having access to the previous determinations. The right arm brachial artery was studied in several longitudinal scans with the probe above the elbow fold. The diameter of the vessel, defined as the distance between the upper echo margin produced by the interface between the lumen and the front wall of the vessel and the upper margin of the echo product from the interface between the lumen and the back wall of the vessel, was measured four times in the peak of the pulsed flow of the spectral curve of the ultrasound, to calculate the mean value. After the initial calculation, a sphygmomanometer sleeve was placed about 3–5 cm above the elbow bend and swollen rapidly at a higher pressure of about 25–30 mmHg compared to the previously measured systolic blood pressure. The pressure was maintained for 5 minutes and at the end of this period, rapid swelling of the sleeve was carried out, leading to reactive hyperemia and measurement of the diameter of the brachial artery at intervals of about 20 seconds for 3 minutes, considering that the maximum expansion value is obtained on average between 60 and 90 seconds. The Flow Mediated Dilation (FMD) value was calculated as the percentage difference between the maximum post-hyperemic diameter reached and the mean basal diameter using the formula: FMD (%) = [(post-hyperemia diameter − basal diameter)/basal diameter] × 100 [25].

### 2.7. Statistical Analysis

Statistical analysis was performed using Stata 14 (StataCorp LLC, College Station, TX, USA). Baseline characteristics were compared using a Student’s *t*-test and chi-square test. A paired *t*-test was used to assess changes of studied variables within each group. A Student’s *t*-test was conducted to compare differences in all variables between groups after the follow-up period. Pearson correlation analysis was performed between the studied variables. A subgroup analysis was conducted in three subgroups according to baseline FLI categories. A box plot was used to show delta changes in FLI at the end of the study, in relation to the presence and degree of NAFLD at baseline. Data are expressed as mean + standard deviation for parametric variables and as median (range) for nonparametric ones, including a 95% confidence interval, whereas categorical parameters are expressed as percentages.

We have analyzed many different outcome variables. It could be argued to correct the p-value for multiple comparisons (i.e., Bonferroni’s correction), but this type of correction is not free of criticism, particularly when those variables are correlated, increasing the number of false negatives, i.e., reducing the statistical power [26]. Instead, we offer the exact p-value, allowing the reader to, eventually, make their own correction based on the number of variables they are interested in.

Yet, we tested the potential confounding effect of gender, age, and years of diabetes (since these are very well-known variables potentially associated with outcome variables), on the difference between the test and control for those studied variables. For that purpose, and for each variable, we built two regression models, one with group (test and control), and the other with group, gender and age, and years of diabetes. We considered the potential confounding bias if the coefficient for the group variable changed more than 25% between both models.

## 3. Results

### 3.1. Baseline Subjects Characteristics

**Figure 1:**
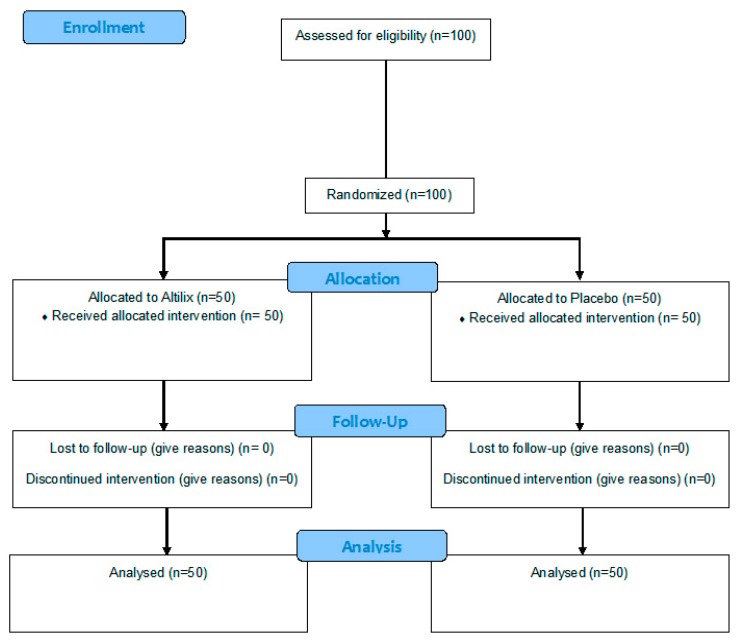
Flow diagram of study.

A total of 100 subjects with MetS were enrolled in this study. Fifty subjects were randomized to Altilix^®^ (26 men and 24 women, mean age 63 ± 9 years) and the other 50 to placebo (28 men and 22 women, mean age 60 ± 10 years). Overall tolerability to the supplements was excellent, and the only uncommon side effect was transient gastrointestinal symptoms (as reported in two subjects under Altilix^®^ and in three subjects under placebo). None of the subjects had to discontinue the supplementation. All participants were the same ethnicity, while all women included in the study were in menopause and ≥50 years of age who have had cessation of menses for at least 1 year. Figure 1 shows a flow diagram of the study sample. 

Table 1 describes baseline characteristics of both groups and there were no significant statistical differences between them.

In no studied model did the inclusion of gender, and age and diabetes produced a change greater than 25%, indicating the absence of important confounding bias derived from age and gender and diabetes.

### 3.2. Cardiometabolic and Liver Parameters

Table 2 shows cardiometabolic and liver parameters, as assessed at baseline and at the end of 6 months follow-up, for subjects under Altilix^®^ or placebo; we also show in this table the comparative percentage change for each parameter between the two study groups. At the end of the study, we found that subjects receiving Altilix^®^, in relation to placebo, had a statistically significant improvement in most of the evaluated parameters, including body weight and BMI (*p* < 0.001 for both), waist circumference (*p* = 0.003), glucose metabolism parameters (HbA1c, HOMA-IR, and HOMA-β, *p* = 0.001 for all), lipid profile (TC, TG, and LDL-C, *p* < 0.001 for all), hepatic enzymes (AST, ALT, AST/ALT ratio, *p* < 0.001 for all), as well as FLI, FMD, and cIMT, (*p* < 0.001 for all).

### 3.3. Clinical and Biochemical Parameters

Clinical and biochemical parameters were also analyzed by FLI subgroups in order to relate them with the presence and degree of NAFLD, as shown in Table 3. At the end of the study, we again found that subjects receiving Altilix^®^, in relation to placebo, had a statistically significant improvement in most of the evaluated parameters. Weight and BMI improved in subjects with NAFLD (*p* = 0.031 for both), while waist circumference improved in those with borderline NAFLD and NAFLD (*p* = 0.013 for both). Glycemic variables (HbA1c, HOMA-IR, and HOMA-β), plasma lipids (TC, TG, and LDL-C), and hepatic enzymes (AST, ALT, and AST/ALT ratio) improved in all the three NAFLD subgroups (mostly *p* < 0.001 for all parameters). In addition, a significant improvement of FLI, cIMT, and FMD was observed in the three NAFLD subgroups (mostly *p* < 0.001 for all parameters).

### 3.4. Fatty Liver Index

**Figure 2:**
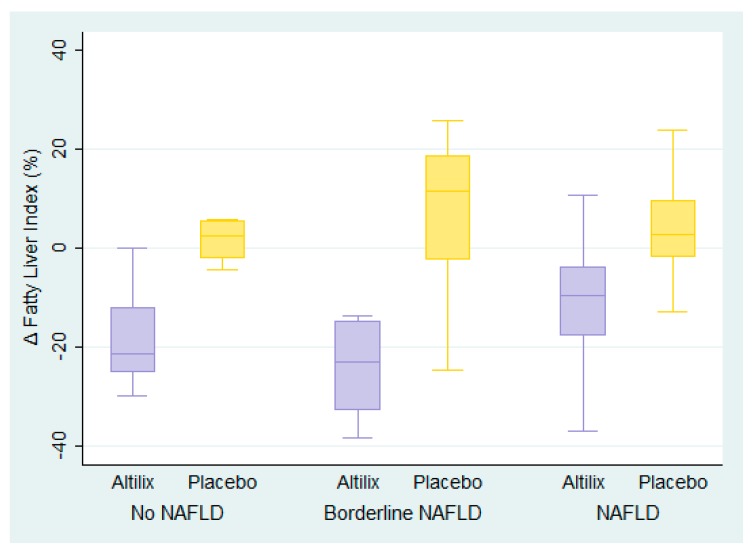
Box plots of fatty liver index (FLI) percentage change at the end of the study in all subjects, according to the baseline presence and degree of NAFLD (*n* = 100).

Box plots of FLI percentage changes at the end of the study in all subjects according to baseline presence and degree of NAFLD are shown in Figure 2. We found a reduction in FLI in subjects receiving Altilix^®^, independent of baseline presence and degree of NAFLD. By contrast, subjects receiving the placebo had null or negative effect.

### 3.5. Cytokine Analysis

Results of the cytokine analysis are depicted in Table 4. Some samples were lost to follow-up and therefore the comparison was performed with the subjects with samples from baseline and after 6 months. A significant reduction in serum levels of ghrelin were found in the subjects receiving Altilix^®^. No differences were found in the rest of inflammatory cytokines analyzed.

### 3.6. Pearson’s Correlation

Pearson’s correlation was performed for each variable between the study groups. Results are displayed in Table 5. Significant correlations were found in the clinical variables, coherent with the results obtained in the bivariate analyses. No significant correlations were observed in the inflammatory cytokine levels.

## 4. Discussion

Literature data evidences that more efficient strategies are urgently needed in order to reduce the epidemiologic burden and adverse outcomes of the cardiometabolic implications of MetS and NAFLD. 

A recent preclinical study reported the impact of flavonol derivatives rich in quercetin and kaempferol on fat storage in the liver. These molecules suppressed some signaling pathways involved in lipogenesis and adipogenesis, as well in the expression of key proteins of lipogenic liver metabolism [27]. Our results point out the presence of such synergistic effects, showing an improvement in anthropometric (body weight, BMI, and waist circumference), glycemic, lipid, and liver parameters in Altilix^®^ (based on chlorogenic acid and its derivatives, and luteolin and its derivatives) supplemented subjects vs. placebo. These beneficial effects were maintained even when we stratified subjects according to the presence and degree of NAFLD. Our findings are in accord with recently published data of a pilot trial, in which an improvement of anthropometric variables after artichoke leaf extract supplementation in NAFLD subjects was present [28]. Other preclinical and clinical studies have demonstrated several health-promoting properties of extracts of *Cynara* spp., such as hypoglycemic, hypocholesterolemic, hypotriglyceridemic, other than antioxidant and hepatoprotective actions [11]. In the present rigorous randomized, double-blind, placebo-controlled study, after 6 months of Altilix^®^ supplementation, we confirmed the beneficial effects on hepatic and cardiometabolic parameters. It may be considered that such results could be due to the impact on oxidative stress state elicited by *Cynara cardunculus* phytochemicals, a key factor in the pathogenesis of NAFLD, as well as on inflammation [11].

Our experimental data, for the first time, show the effect of artichoke extract on the improvement of two early atherosclerotic markers: cIMT and FMD, evidencing the clinical importance of the treatment as well as its nutraceutical properties on vascular function and remodeling, including beneficial action on the cardiovascular system and hepatoprotective activity [10]. Firstly, after 6 months of Altilix^®^ supplementation, we found a significant improvement in endothelial function, and subsequently, we confirmed a preliminary observation of a beneficial effect of artichoke juice on brachial FMD, in a small group of hyperlipidemic subjects [29]. 

The measured beneficial effect of cIMT in Altilix^®^ supplemented in comparison with placebo, is consistent with the positive influence of the treatment on LDL-cholesterol, and on the reduction in cardiovascular risk for MetS subjects. It is well known that elevated LDL-cholesterol levels are the major risk factor for coronary heart disease and, together with high cholesterol and triglycerides, are the main risk factors for atherosclerosis [30]. Research on animal models showing that artichoke extract may prevent the development of atherosclerotic plaques [31] has commented that the results are due to an antioxidant effect mediated by the reduction of LDL oxidation [32] and the inhibition of cholesterol synthesis [33].

We also showed that there is not a link between the degree of steatosis and improvement in cardiometabolic parameters; so, we hypothesize that Altilix^®^ could act as a hepatic detoxifying agent with which supplementation could be considered very useful in the prevention of hepatic complications of MetS, particularly in NAFLD patients. This project could benefit from stratified analyses, based on variables such as NAFLD or other. Nevertheless, the sample sizes would decrease to get enough statistical power. Thus, we prefer to maintain the comparisons between the whole test and control groups; also, it should be considered that the test and control groups were randomly assigned.

In fact, we found in subjects from the Altilix^®^ supplemented vs. placebo groups, regarding MetS comorbidities, a significant improvement in glucose metabolism parameters (including HbA1c, insulin resistance, and pancreatic β-cell function), in agreement with some in vitro and in vivo literature data [34,35,36]. In particular, among these, some in vivo studies with oral daily administration of *Cynara cardunculus* (L.) subsp. *scolymus* Hayek extract showed glycemic-lowering effects. Other studies reported in 39 overweight subjects, that the intake of tablets containing extract of artichoke during meals, resulted in a significant reduction of the HOMA index [34]. Whereas, in vitro studies evidenced the role of chlorogenic acid on glucose regulation—antagonist in glucose transport, inhibitor of α-amylase and α-glucosidase, and therefore in the post-prandial glucose blood concentration [34,35,36]. We also found, after Altilix^®^ supplementation, an improvement in both HOMA-IR (marker of insulin resistance), a marker useful in identifying individuals with metabolic NAFLD [37] and HOMA-β (marker of pancreatic β-cell function). 

Regarding the effect on cardiovascular/cardiometabolic diseases, our data concerning a significant reduction in TC, TG, and LDL-cholesterol, together with some not significant modifications in HDL-cholesterol levels, are also in line with previous reported effects of artichoke on lipid profile [38] as well as on the inhibition of cholesterol synthesis exerted by luteolin, one of the components of Altilix^®^ [33,39]. However, this lipid-lowering effect could be, in turn, attributed to the presence in Altilix^®^ of chlorogenic acid, which is associated with a direct action on the liver, the excretion of biliary salts, and acids rich in cholesterol [34,40]. Therefore, our findings, proposing a direct effect of artichoke extract on lipids affecting liver-related parameters, particularly TG, result in being, again, of huge clinical value, since the accumulation of TG in hepatocytes is largely involved in NAFLD development and progression [41]. 

To reinforce the values of Altilix^®^ supplementation in improving hepatic function in NAFLD patients, there is in our data the enhanced AST/ALT ratio, a reduction in both AST and ALT and FLI serum levels after 6 months of supplemented subjects compared with the placebo group, suggesting that subjects at high risk of developing hepatic complications of MetS could benefit from the mentioned supplementation. Previous evidence has shown, in fact, a possible activity of serum ALT as a predictor factor for general health, independent of the presence of liver disease, and particularly useful when liver disease is a component of MetS [42]. 

Artichoke leaf extract has been proven, also, for its anti-inflammatory effects in non-alcoholic steatohepatitis induced in animal models [43]. In addition, compounds derived from artichoke, too, have been previously described to act in the anti-inflammatory process, even in subjects with NAFLD [15,28]. These hepatoprotective effects may be surely ascribed to the antioxidant effects of phenols present in artichoke leaves. In fact, it may be considered that the intake of *Cynara cardunculus* extract can lead to the removal of dangerous toxins, facilitation of bile production, assisting fat digestion and significantly preventing the lipid peroxidation process in cell membranes of liver tissues, as well as oxidative damage in hepatocyte membranes [44,45]. Finally, a significant rise of the serum levels of ghrelin was observed in the subjects that received the Altilix^®^ supplement. Lower levels of this hormone have been previously related to weight gain and adiposity, since it physiologically increases during fasting and decreases after food intake and has also a role in energy expenditure [46]. Its levels have also been reported to be elevated by drugs such as Liraglutide [47]. A previous study testing the effects in rodents of an extract of *Cosmos caudatus* Kunth leaf, rich in chlorogenic acid, also showed an increase in ghrelin levels, as well as an improvement in lipid and obesity-related variables [48].

It is well known that concentrations of IL-6 are drastically increased during inflammatory conditions [49,50]. We suppose that one of the reasons for the remarkable difference among the Altilix^®^ and placebo groups can be the increased inflammation associated with higher BMI and increased waist circumference in the placebo group that is further associated with visceral fat and its mediators in the inflammation process. Also, some other mediators, such as higher number of smokers in the placebo group, can be associated with higher inflammation in this group. The same reasons, at least in part, might explain that ghrelin is reduced in the placebo group, as it is known that plasma ghrelin levels decrease after a meal is consumed and in conditions of obesity, as well as that ghrelin levels are negatively correlated with BMI and insulin resistance [51]. Yet, we cannot exclude the possibility that Altilix^®^ interferes in the mechanism of secretion of ghrelin and in the regulation of gastric emptying. In addition, in the placebo group, the cIMT increased after 6 months which also might, in part, explain lower levels of ghrelin as it is known that MetS is a predisposing factor to arterial stiffness, that persistent MetS circumstances can deteriorate the arterial stiffness severity, and that low concentrations of plasma ghrelin are meticulously connected to arterial stiffness [52].

Such findings indicate the importance of a multifactorial approach on several components of MetS simultaneously, including lifestyle changes and promotion of non-smoking. It seems that different ingredients of the natural supplement used in the present study could have such multifactorial effects that can be of particular benefit for subjects with MetS.

The strengths of the study design are the inclusion of the placebo group, of the rigorous randomization, the double-blinded research, the high adherence to the treatment, as well as the blinded measurements of all parameters, including cIMT and FMD. This finding is of considerable clinical value, and we observed a general improvement in all groups. An improvement in cIMT and FMD in subjects with and without NAFLD in our study remains a valid therapeutic option, but the possible mechanisms of action on cardiovascular risk remains to be clarified. Studies with longer follow-ups are still necessary to confirm these effects. Also, the presented results have shown significant clinical effects in a real-world setting. Conversely, potential limitations may include the relatively short time frame of 6 months and the avoided acquisition of liver biopsy. However, this may be considered only an apparent limitation from both a clinical and ethical point of view; a liver biopsy could not be justified. On the other hand, the use of FLI for evaluating the presence and degree of NAFLD, as done in the present study, has been shown over the years very useful, since FLI is consistently able to detect unrecognized liver disease in the general population [53]. Also, the American Association for the Study of Liver Diseases approves non-invasive methods, such as the assessment of serum markers, for screening liver dysfunction in subjects with metabolic risk factors, while liver biopsy has to be considered for disease confirmation only [54]. 

## 5. Conclusions

Altogether, our data demonstrate that Altilix^®^ supplementation strongly improved quality of life of both MetS and NAFLD patients, ameliorating some key biomarkers obtained through a very rigorous randomized, double-blind, placebo-controlled study. Therefore, we suggest Altilix^®^ supplementation as a valid and safe approach in the prevention and management of cardiometabolic and hepatic alterations, such as NAFLD and MetS.

## Figures and Tables

**Table 1:** Baseline characteristics of all subjects included in the study (*n* = 100).

Variable	Altilix (*n* = 50)	Placebo (*n* = 50)	*p*-Value
Age (years), mean ± SD	63.0 ± 9.32	60.2 ± 10.3	0.161 ^a^
Women, *n* (%)	24 (48)	22 (44)	0.688 ^b^
Smoking habit, *n* (%)	9 (18)	15 (30)	0.160 ^b^
Family history of cardiovascular diseases, *n* (%)	33 (66)	31 (62)	0.677 ^b^
Diabetes duration (years), mean ± SD	8.40 ± 7.05	7.24 ± 8.03	0.449 ^a^
Hypertension, *n* (%)	41 (82)	38 (76)	0.461 ^b^
Dyslipidemia, *n* (%)	42 (84)	39 (78)	0.444 ^b^
Obesity, *n* (%)	27 (54)	32 (64)	0.309 ^b^

^a^ Student’s *t*-test; ^b^ Pearson’s chi-square test.

**Table 2:** Cardiometabolic parameters at baseline and at the end of the study (*n* = 100).

Parameters	Altilix (*n* = 50)		Placebo (*n* = 50)		% Change (*n* = 100)			
	Baseline	6 Months	Baseline	6 Months	Altilix	Placebo	Difference (95% CI)	*p*-Value ^a^ (between Groups)
Weight (kg)	82.63 ± 16.4	80.06 ± 15.5	87.37 ± 16.5	86.88 ± 17.0	−3.00	−0.60	−2.40 (−3.79, −1.01)	<0.001
BMI (kg/m^2^)	31.04 ± 5.15	30.08 ± 4.97	32.10 ± 5.80	31.89 ± 5.78	−3.01	−0.60	−2.41 (−3.80, −1.03)	<0.001
Waist circumference (cm)	106.6 ± 12.6	103.1 ± 11.7	111.2 ± 12.5	110.7 ± 14.0	−3.21	−0.45	−2.76 (−4.55, −0.96)	0.003
HbA1c (%)	7.28 ± 1.18	6.51 ± 0.88	7.24 ± 1.44	7.42 ± 1.53	−0.77	0.18	−0.95 (−1.22, −0.67)	<0.001
HOMA-IR	4.005 ± 2.85	3.049 ± 1.99	5.157 ± 6.32	5.763 ± 6.39	−21.10	22.0	−43.11 (−51.87, −34.35)	<0.001
HOMA-β	80.65 ± 52.8	96.98 ± 54.7	87.37 ± 96.3	61.99 ± 60.9	16.3	−25.4	41.70 (17.58, 65.83)	0.001
Total cholesterol (mg/dL)	191.6 ± 41.1	165.2 ± 42.2	177.8 ± 36.8	188.1 ± 40.7	−13.6	6.03	−19.59 (−23.71, −15.47)	<0.001
Triglycerides (mg/dL)	154.1 ± 102	115.6 ± 46.2	143.6 ± 68.3	166.4 ± 79.25	−17.2	17.9	−35.14 (−44.83, −25.45)	<0.001
LDL-cholesterol (mg/dL)	116.6 ± 34.4	97.79 ± 36.5	105.0 ± 32.9	112.8 ± 34.0	−15.9	8.89	−24.79 (−31.43, −18,16)	<0.001
HDL-cholesterol (mg/dL)	48.82 ± 11.5	48.71 ± 11.3	47.42 ± 11.1	47.31 ± 12.1	0.80	0.26	0.54 (−4.83, 5.91)	0.842
Fatty Liver Index	59.55 ± 26.7	50.27 ± 27.2	62.39 ± 25.4	64.50 ± 26.1	−17.7	4.17	−21.83 (−27.39, −16.27)	<0.001
AST	20.68 ± 8.35	18.56 ± 11.3	19.90 ± 5.82	21.72 ± 6.11	−10.3	10.3	−20.54 (−26.87, −14.22)	<0.001
ALT	26.96 ± 13.7	19.84 ± 9.65	22.26 ± 11.3	26.62 ± 11.9	−23.3	23.7	−47.06 (−54.97, −39.13)	<0.001
AST/ALT	0.827 ± 0.22	0.978 ± 0.26	0.996 ± 0.29	0.904 ± 0.31	21.1	−8.66	29.71 (19.66, 39.77)	<0.001
Flow-mediated dilation (%)	21.53 ± 16.1	26.26 ± 12.4	16.85 ± 14.5	10.93 ± 6.85	4.64	−5.91	10.56 (5.00, 16.12)	<0.001
Carotid IMT (mm)	0.922 ± 0.16	0.598 ± 0.14	0.862 ± 0.17	0.892 ± 0.18	−33.4	6.03	−39.48 (−47.98, −30.97)	<0.001

All values expressed as mean ± standard deviation and percent change; ^a^ Student’s *t*-test. HOMA: homeostatic model assessment; LDL: low-density lipoprotein-cholesterol; HDL: high-density lipoprotein-cholesterol; AST: aspartate aminotransferase; ALT: alanine aminotransferase; IMT: intima-media thickness.

**Table 3:** Cardiometabolic parameters at baseline and at the end of the study, in relation to the presence and degree of non-alcoholic fatty liver disease (NAFLD) (*n* = 100).

Parameters	Without NAFLD (*n* = 22)				With Borderline NAFLD (*n* = 20)				With NAFLD (*n* = 58)			
	Altilix (*n* = 12)	Placebo (*n* = 10)	Difference (95% CI)	*p*-Value ^a^ (between Groups)	Altilix (*n* = 10)	Placebo (*n* = 10)	Difference (95% CI)	*p*-Value ^a^ (between Groups)	Altilix (*n* = 28)	Placebo (*n* = 30)	Difference (95% CI)	*p*-Value ^a^ (between Groups)
Weight (kg)	−3.3	−0.9	−2.49 (−5.32, 0.34)	0.082	−2.7	0.4	−3.02 (−6.32, 0.27)	0.070	−3.0	−0.8	−2.14 (−4.08, −0.20)	0.031
BMI (kg/m^2^)	−3.4	−0.9	−2.54 (−5.37, 0.28)	0.082	−2.7	0.4	−3.02 (−6.32, 0.27)	0.070	−3.0	−0.8	−2.14 (−4.08, −0.20)	0.031
Waist circumference (cm)	−1.9	−1.2	−0.70 (−5.51, 4.11)	0.764	−4.3	−0.2	−4.09 (−7.18, −0.99)	0.013	−3.4	−0.3	−3.10 (−5.51, −0.68)	0.013
HbA1c (%)	−0.7	0.2	−0.87 (−1.67, −0.08)	0.033	−1.0	−0.3	−0.96 (−1.64, −0.27)	0.009	−0.7	0.2	−0.96 (−1.28, −0.64)	<0.001
HOMA-IR	−19.7	13.2	−32.94 (−43.77, −22.11)	<0.001	−24.7	38.5	−63.13 (−87.23, −39,03)	<0.001	−20.1	19.3	−39.35 (−49.97, −28.72)	<0.001
HOMA-β	11.1	−4.0	15.02 (8.14, 21.91)	0.002	11.8	−15.6	26.80 (10.58, 43.02)	0.002	19.0	−31.7	50.68 (15.13, 86.23)	0.006
Total cholesterol (mg/dL)	−16.8	3.0	−19.84 (−30.13, −9.55)	<0.001	−14.9	6.5	−21.33 (−28.07, −14.59)	<0.001	−11.7	6.8	−18.48 (−24.25, −12.71)	<0.001
Triglycerides (mg/dL)	−21.5	7.0	−28.52 (−45.40, −11.65)	0.002	−12.7	13.5	−26.19 (−38.32, −14.05)	<0.001	−17.0	22.7	−39.66 (−54.67, −24.65)	<0.001
LDL-cholesterol (mg/dL)	−16.0	7.4	−23.40 (−41.90, −4.90)	0.016	−21.1	6.8	−27.90 (−35.98, −19.82)	<0.001	−14.0	10.0	−24.04 (−33.37, −14.71)	<0.001
HDL-cholesterol (mg/dL)	−4.7	−2.7	−2.01 (−15.33, 11.30)	0.755	4.4	9.8	−5.32 (−21.01, 10.36)	0.485	1.7	−1.9	3.59 (−2.26, 9.44)	0.224
Fatty Liver Index	−21.7	5.8	−27.47 (−41.46, −13.49)	<0.001	−24.2	7.1	−31.28 (−43.07, −19.49)	<0.001	−13.6	2.7	−16.26 (−23.38, −9.14)	<0.001
AST	−10.9	6.9	−17.80 (−32.08, −3.53)	0.017	−24.3	15.3	−39.63 (−48.81, −30.46)	<0.001	−4.96	9.73	−14.69 (−23.28, −6.10)	0.001
ALT	−23.0	15.9	−38.92 (−57.16, −20.67)	<0.001	−30.7	46.4	−77.11 (−87.77, −66.45)	<0.001	−20.8	18.8	−39.61 (−49.83, −29.38)	<0.001
AST/ALT	20.6	−6.5	27.11 (6.23, 48.00)	0.014	9.6	−21.1	30.71 (24.12, 37.31)	<0.001	25.3	−5.2	30.56 (15.26, 45.86)	<0.001
Flow-mediated dilation (%)	−0.5	−8.7	8.19 (−10.69, 27.06)	0.376	4.9	−2.1	6.96 (3.23, 10.69)	0.001	6.8	−6.3	13.04 (6.24, 19.83)	<0.001
Carotid IMT (mm)	−28.8	16.3	−45.05 (−66.09, −24.01)	<0.001	−35.3	6.7	−41.96 (−57.91, −26.01)	<0.001	−34.8	2.4	−37.19 (−48.74, −25.65)	<0.001

All values expressed as percent change; a: Student’s *t*-test.

**Table 4:** Results of cytokine analysis of the patients of the study (*n* = 50).

Cytokine	Altilix (*n* = 25)			Placebo (*n* = 25)			% Change (*n* = 50)		
	Baseline	6 Months	*p-*Value ^a^ (within Group)	Baseline	6 Months	*p-*Value ^a^ (within Group)	Altilix	Placebo	*p-*Value ^b^ (between Groups)
IL-1 (pg/mL)	4.80 ± 1.70	5.03 ± 2.20	0.504	3.32 ± 2.23	2.69 ± 0.79	0.093	6.29	−3.75	0.263
IL-6 (pg/mL)	23.13 ± 8.79	25.86 ± 10.66	0.078	13.66 ± 7.98	15.30 ± 13.26	0.448	16.41	10.99	0.660
GLP-1 (pg/ml)	38.33 ± 45.25	38.11 ± 48.14	0.984	44.74 ± 47.36	61.21 ± 54.20	0.093	−0.22	16.47	0.253
RANTES (pg/mL)	1331.51 ± 1074.32	1673.34 ± 1137.85	0.232	1557.48 ± 1140.62	1879.08 ± 1233.35	0.244	112.67	74.45	0.431
Ghrelin (pg/mL)	1984.07 ± 917.78	2739.52 ± 1840.74	0.024	2521.72 ± 981.29	2481.26 ± 1470.86	0.908	42.00	9.91	0.134
Leptin (pg/mL)	14171 ± 9287.93	15363.12 ± 9726.65	0.679	14529.97 ± 8322.74	12360.38 ± 9633.23	0.414	73.39	48.20	0.590
Resistin (pg/mL)	2745.39 ± 847.26	2525.14 ± 1312.40	0.482	2499.69 ± 1068.48	2689.30 ± 1017.73	0.522	1.77	30.73	0.136

All values expressed as mean ± standard deviation. Change expressed as percentage except GLP-1 which is absolute change; ^a^ Student’s paired *t*-test; ^b^ Student’s *t*-test.

**Table 5:** Pearson’s correlation analysis of the variables between the study groups (*n* = 100).

Variable	Correlation Coefficient (r^2^)	*p*-Value
Weight	0.3276	<0.001
BMI	0.3294	<0.001
Waist circumference	0.2947	<0.001
HbA1c	0.5720	<0.001
HOMA-IR	0.7708	<0.001
HOMA-β	0.3910	0.001
Total cholesterol	0.6917	<0.001
Triglycerides	0.5899	<0.001
LDL-cholesterol	0.6034	<0.001
HDL-cholesterol	−0.0203	0.842
Fatty Liver Index	0.6183	<0.001
AST	0.5456	<0.001
ALT	0.7659	<0.001
AST/ALT	−0.5096	<0.001
Flow-mediated dilation	−0.3558	<0.001
Carotid IMT	0.6811	<0.001
IL-1	−0.1509	0.263
IL-6	−0.0613	0.660
GLP-1	0.1632	0.253
RANTES	−0.1064	0.431
Ghrelin	−0.2104	0.134
Leptin	−0.0723	0.590
Resistin	0.2000	0.136

Correlation analysis of percentage change of each variable with the group variable, with Group 1 being the Altilix^®^ group and Group 2 the placebo group.
